# CCL25/CCR9 Interactions Regulate the Function of iNKT Cells in Oxazolone-Induced Colitis in Mice

**DOI:** 10.1371/journal.pone.0100167

**Published:** 2014-06-17

**Authors:** Siying Zhu, Yuntao Bing, Xiaobing Wang, Qiao Yu, Yipeng Wang, Shufang Xu, Lu Song, Xintao Wang, Bing Xia, Youqing Zhu, Rui Zhou

**Affiliations:** 1 Department of Gastroenterology/Hepatology, Zhongnan Hospital of Wuhan University, Wuhan, Hubei, P. R. China; 2 The Hubei Clinical Center & Key Laboratory of Intestinal & Colorectal Diseases, Wuhan, Hubei, P. R. China; CNRS, France

## Abstract

**Background:**

Natural killer T (NKT) cells share phenotypic and functional properties with both conventional natural killer cells and T cells. These cells might have an important role in the pathogenesis of ulcerative colitis (UC). The interaction of chemokine ligand 25 (CCL25) with chemokine receptor 9 (CCR9) is involved in gut-specific migration of leukocytes and induces regulatory T cells (Tregs) to migrate to the intestine in chronic ileitis.

**Methodology/Findings:**

In UC patients, NKT receptor CD161, CCL25, and CCR9 expression levels were evaluated by qRT-PCR. A murine model of oxazolone-induced colitis was induced in BALB/c mice. The mRNA levels of NK1.1, CCL25 and CCR9, and pro-inflammatory cytokines in mice were evaluated. The CCR9 expression on Type I or invariant NKT (iNKT) cells, and the iNKT cells chemotaxis are observed according to flow cytometry. NKT receptor CD161, CCL25 and CCR9 expression levels were significantly increased in UC patients. And, the mRNA expression levels of NK1.1, CCL25 and CCR9 were increased in oxazolone-induced colitis in mice. The production of pro-inflammatory cytokines was significantly increased, especially interleukin 4 (IL-4), IL-10 and IL-13. We observed significantly increased CCR9 expression on iNKT cells. Furthermore, we found an increased iNKT population and enhanced chemotaxis during oxazolone-induced colitis.

**Conclusions/Significance:**

Our study suggests that CCL25/CCR9 interactions may promote the induction and function of iNKT cells during oxazolone-induced colitis. These findings may have important implications for UC treatment and suggest a role for CCR9 inhibitors.

## Introduction

Ulcerative colitis (UC) and Crohn’s disease (CD) are clinical subtypes of inflammatory bowel disease (IBD) and are chronic, relapsing immune-mediated disorders of the gastrointestinal tract with unknown etiology [1]. Nevertheless, UC and CD differ from one another both clinically and pathologically [2]. UC is characterized by a T-helper type 2 (Th2) immune responses with contiguous mucosal inflammation in the rectum and colon that cause epithelial barrier dysfunction and lead to ulceration [3]. There are several murine models of mucosal inflammation that mimic human IBD, including a model of hapten-induced colitis in which oxazolone (4-ethoxymethylene-2-phenyl-2-oxazoline-5-one) is delivered intrarectally to rodents. This model is driven by the production of Th2 cytokines and reproduces many UC features [4], [5].

Natural killer T (NKT) cells share phenotypic and functional properties with both conventional natural killer cells and T cells. NKT cells recognize the foreign or microbial lipid antigens presented by the non-classical major histocompatibility complex (MHC) molecule CD1d [6]. There are distinct NKT-cell subsets and other types of T cell that resemble NKT cells. NKT cells include CD1d-dependent NKT cells (type I and II) and CD1d-independent NKT-like cells [7]. CD1d-dependent NKT cells are divided into 2 subsets based on differences in T cell receptor (TCR) characteristics [8]. Type I or invariant NKT (iNKT) cells are composed of an invariant TCR α-chain (Vα14-Jα18 in mice and Vα24-Jα18 in humans) paired with a limited set of TCR β-chain. These cells are present in both human and mouse intestines [9]. iNKT cells recognize the marine sponge-derived glycolipid α-galactosylceramide (α-GalCer) in mureine and humans. However, Type II NKT cells are other populations of CD1d-dependent NKT cells, which respond to lipid antigens are broadly. Type II NKT cells exhibit much more TCR sequence diversity and do not respond to α-GalCer, compared to iNKT cells [6]. The most commonly described subset is the iNKT subset [8]. iNKT cells most likely play an important role in the pathogenesis of UC and asthma [10]–[12].

Chemokine ligand 25 (CCL25, TECK) is highly expressed by the intestinal epithelium and thymus, and regulates trafficking of gut-specific memory/effector T cells via upregulation of the integrin homing receptor α47 and chemokine receptor 9 (CCR9) [13], [14]. CCR9 has been associated with IBD and other inflammatory disorders of the intestine, such as celiac disease and primary sclerosing cholangitis [15]–[17]. CCX282-B is an orally bioavailable CCR9 antagonist that can delay disease progression in moderate to severe Crohn’s Disease patients [18]. However, the role of CCL25/CCR9 interactions in the regulation of NKT cells during colitis has not been studied. In the present study, we evaluated the role of CCL25/CCR9 interactions in the regulation of NKT cells in a model of oxazolone-induced colitis.

## Materials and Methods

### Ethics Statement

All specimen study was approved by the Medical Ethical Committee of the Zhongnan Hospital of Wuhan University and conducted according to the principles expressed in the Declaration of Helsinki. A written informed consent was obtained from all patients and healthy individuals participating in this study. The individual in this manuscript has given written informed consent (as outlined in PLOS consent form) to publish these case details. All animal procedures were performed in strict accordance with the recommendations in the Guide for the Care and Use of Laboratory Animals of Wuhan University. The protocols were approved by the Committee on the Ethics of Animal Experiments of Wuhan University. All of the surgeries were performed under sodium pentobarbital anesthesia, and all efforts were made to minimize suffering.

### Patients and Specimens

For this study, 10 consecutive UC patients (5 men and 5 women, aged 21–79 years) were enrolled from January 2012 to January 2013. The diagnosis of UC was based on clinical, endoscopic, radiological and histological findings according to the Lennard-Jones (1989) criteria [19]. The level of disease activity was assessed according to the Truelove and Witt activity index [20]. A total of 10 age- and sex-matched healthy controls were also enrolled in the study. The controls were healthy volunteers unrelated to the patients. Colonic biopsy specimens were obtained from the UC patients and healthy controls by elective colonoscopy.

### Animals

Specific pathogen-free 6- to 8-wk-old BALB/c male mice weighing 20–25 g were purchased from the Experimental Animal Center of Wuhan University. All mice were maintained in plastic cages with free access to pellet food and water. The animals were housed at 21±2°C and kept on a 12-h light/dark cycle. The animals were acclimated to these conditions for at least 5 days before the start of the experiment. Animals were randomly allocated to three groups using a random numbers table. Each group contained 15 animals. Animals were humanely sacrificed if they reached a predetermined experimental end point (i.e., loss of ≥20% body weight and significant deterioration of body conditions).

### Induction of Colitis

Oxazolone (4-ethoxymethylene-2-phenyl-2-oxazolin-5-one) was obtained from Sigma-Aldrich (St. Louis, MO, USA). Oxazolone (OXA) colitis was induced as previously described [21]. To pre-sensitize mice, a 2×2 cm field of the abdominal skin was shaved, and 150 µl of a 3% (w/v) solution of OXA in 100% ethanol (ETOH) was applied. Seven days later, 150 µl of 2% OXA in 50% ETOH was administered intrarectally with a polyethylene catheter inserted 3 cm into the colon of the OXA mice. The ETOH mice were treated intrarectally with 150 µl of 50% ETOH alone. The control mice were treated without OXA or ETOH. The mice were autopsied 2 days post-oxazolone treatment and sacrificed by cervical dislocation under anesthesia. All of the surgeries were performed under sodium pentobarbital anesthesia, and efforts were made to minimize suffering.

### Evaluations of Colitis Severity

Mice were examined daily for body weight, stool consistency, hematochezia and rectal bleeding to grade the clinical colitis severity. The clinical disease activity index (DAI) was determined based on weight loss, stool consistency, hematochezia and rectal bleeding using standard protocols [22], [23], as shown in Table 1. The animal survival rate was monitored during the experiment, and colon length was measured after the autopsy.

**Table 1:** Scoring system for disease activity index (DAI) in the study.

Score	Weight loss (%)	Stool consistency	Hematochezia	Rectal bleeding
0	0	Normal	None	Absent
1	1–10	Loose stool	Hemaoccult positive	Present
2	11–20	Diarrhea	Gross blood	N/A
3	≥20	N/A	N/A	N/A

### Histological Assessment of Colitis

Colons were collected on day 2 post-oxazolone administration and fixed in 10% neutral buffered formalin. After paraffin embedding, 5-µm sections were cut and stained with hematoxylin and eosin. The stained sections were examined for evidence of colitis by a blinded reviewer. The histology damage score was calculated on a 12-point scale: loss of architecture, 0–3; inflammatory infiltrate, 0–3; goblet cell depletion, 0 or 1; ulceration, 0 or 1; edema, 0 or 1; muscle thickening, 0–2; and presence of crypt abscesses, 0 or 1 [24].

### Myeloperoxidase (MPO) Assay

MPO activity was assayed according to the MPO assay kit manufacturer’s instructions (Jiancheng BioEngineering, Nanjing, China). Briefly, MPO is an enzyme found in granulocytes. We assessed MPO activity using a kinetic assay in which H_2_O_2_ is degraded by the MPO released from the samples of colon [25]. The change in absorbance at 460 nm was measured with a Life Science UV/Vis Spectrophotometer DU 530 (Beckman Coulter, USA). The data are presented as units per milligram of tissue, where 1 unit is equal to the amount of MPO required to degrade 1 µmol of H_2_O_2_ per minute at 25°C.

### Quantitative Real-time Polymerase Chain Reaction (qRT-PCR)

RNA was extracted from colonic biopsy specimens and colon tissue samples using TRIzol reagent (Invitrogen, USA) according to the manufacturer’s protocol. Total RNA quantification was performed with a Nanodrop™ spectrophotometer (Thermo Scientific, USA). cDNA was synthesized using an iScript™ cDNA synthesis kit (Bio-Rad, USA). DNA amplification was performed with an iQ5 quantitative PCR System (Bio-Rad, USA) and SYBR® Green Master Mix (Toyobo, Japan) using 50 ng RNA. CD161, CCL25 and CCR9 mRNAs were quantified from the colonic biopsy specimens of UC patients and healthy controls. β-actin was used to normalize expression. For the colon tissue samples from mice, NK1.1, CCL25 and CCR9 mRNAs were quantified using β-actin mRNA for normalization. The gene-specific primers are listed in Table 2. The cycle threshold (Ct) indicated the fractional cycle number at which the PCR product was first detected above a fixed threshold. The relative mRNA levels were determined using the 2^−ΔΔCT^ method.

**Table 2:** The gene-specific primer list in the study.

Primer names	Primer sequences (5′-3′)
Human:	
CD161-F	TAATGCCACCTTCCTCTG
CD161-R	GAATAATCCCAGCACAGC
CCL25-F	CCACCACAACACGCAGAC
CCL25-R	GATGGAGCCCAGAAATGAG
CCR9-F	TTCCGCTTATTCCTTGGT
CCR9-R	CTAGGGAGCAGACAGACG
β-actin-F	TCAGCAAGCAGGAGTATG
β-actin-R	GTCAAGAAAGGGTGTAACG
Mice:	
NK1.1-F	TGGATTGCTGTTTCCGTCTG
NK1.1-R	TATTGTCAACCCACCACCTT
CCL25-F	CCAAGGTGCCTTTGAAGACT
CCL25-R	TCCTCCAGCTGGTGGTTACT
CCR9-F	CCAGGAAATCTCTGGTCTGC
CCR9-R	CTGTGGAAGCAGTGGAGTCA
β-actin-F	GCTGTATTCCCCTCCATCGT
β-actin-R	GCCATGTTCAATGGGGTACT

### ELISA Assays

Tumor necrosis factor alpha (TNF-α), interferon gamma (IFN-γ), interleukin 17 (IL-17), IL-14, IL-13 and IL-10 were assayed by antibody sandwich ELISA in colon lysates using a mouse ELISA immunoassay kit (Beijing Dakewe Biotech Co. LTD., China). Total protein was extracted from tissues using a Total Protein Extraction Kit (Beijing Tiangen Biotech Co. LTD., China) in accordance with the manufacturer’s protocol.

### Isolation of Lamina Propria Lymphocytes (LPLs)

LPL suspensions were obtained as previously described [26]. The colons were flushed and opened longitudinally. To remove intra-epithelial lymphocytes and epithelial cells, intestinal pieces were incubated in HBSS without Ca^2+^/Mg^2+^ supplemented with 2 mM EDTA, 10 mM HEPES, 5% FCS and 1 mM DTT twice for 15 min at 37°C with shaking at 250 rpm. The intestinal pieces were digested in HBSS with Ca^2+^/Mg^2+^, 5% FCS, 1.5 mg/ml Collagenase VIII (Sigma-Aldrich, USA) and 0.1 mg/ml DNase I (Thermo Scientific, USA) for 45 min at 37°C with shaking at 250 rpm. The LPLs were purified using 40% and 80% Percoll gradients (Sigma-Aldrich, USA) by centrifuging at 1,000×g for 20 min at 20°C without brakes. The LPLs were washed and resuspended in fluorescence-activated cell sorting (FACS) buffer or culture medium (RPMI 1640 containing 5% FCS).

### Flow Cytometry

All staining procedures were performed in Staining Buffer (eBioscience, USA) at 4°C. Single-, double-, or triple-color flow cytometric analyses were performed on a FACS Calibur (BD Biosciences, USA). The data were analyzed using FlowJo software version 7.6.1 (Treestar, Ashland, OR, USA). The peridinin chlorophyll protein-Cy5.5 (PerCP-Cy5.5)-conjugated anti-TCRβ antibody (eBioscience, USA) and phycoerythin (PE)-conjugated α-GalCer-loaded CD1d tetramer (ProImmune, UK) were used as cell surface markers of iNKT cell subpopulations [12]. Additionally, fluorescein isothiocyanate (FITC)-conjugated anti-CCR9 antibody (eBioscience, USA) was used for surface receptor staining. For each marker, the threshold of positivity was defined beyond the nonspecific binding observed in the presence of a relevant isotype control antibody.

### In vitro Chemotaxis Assays

The chemokine-dependent migration of LPLs was measured using an *in vitro* 2-chamber migration assay with Transwell Boyden chambers (BD Biosciences, USA) followed by flow cytometry analysis [27]. The chemotaxis assays were performed in pre-warmed migration buffer (RPMI 1640 containing 1% FCS). Primary LPLs were seeded at a density of 1×10^5^ per well into the upper chamber. A total of 600 µl of migration buffer alone or supplemented with 500 ng/ml recombinant mouse CCL25 protein (Reprokine Research Immunity, USA) was added to the lower chamber. The chambers of cells were incubated for 6 hours at 37°C in an incubator with 5% CO_2_. The cells in the lower chambers were centrifuged, fixed in 500 µl Fixation Buffer (eBioscience, USA) and counted using a FACS Calibur (BD Biosciences, USA). The results were expressed as a chemotactic index (CI), which is the ratio between the number of migrating cells in the sample and in the medium control.

### Statistical Analysis

All data are presented as the mean ± SEM or SD. The statistical significance of differences between experimental groups was analyzed using one-way analysis of variance (ANOVA) for overall comparisons or Student’s unpaired *t*-test for individual comparisons. Differences with *p*<0.05 were considered significant. The correlations of CD161 or NK1.1 expression with the CCL25 and CCR9 expression levels, and the correlation between CCL25 and CCR9 were tested using Pearson’s correlation coefficient. Statistical analysis was conducted using SPSS 17.0 software (SPSS for Windows version 17.0, USA).

## Results

### Increased Expression of CD161, CCL25 and CCR9 in UC Patients

**Figure 1:**
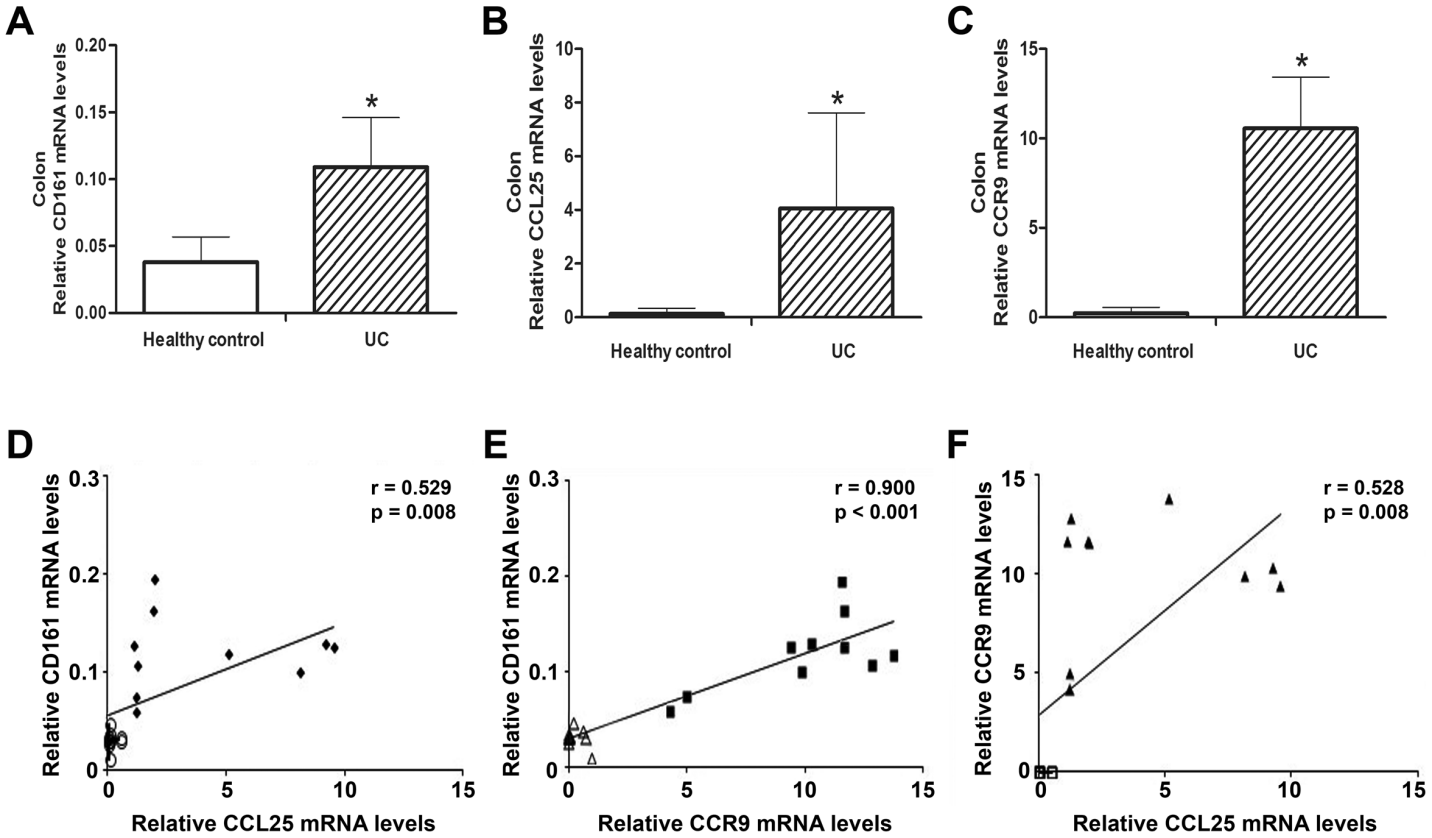
Expression of CD161, CCL25 and CCR9 in UC and healthy controls. The mRNA levels of CD161 (**A**), CCL25 (**B**) and CCR9 (**C**) were significantly increased in the UC patients compared to the healthy controls. n = 10 per group, *p<0.05 versus the healthy controls. **D and E.** CD161 mRNA expression was positively correlated with CCL25 mRNA and CCR9 mRNA expression. **F.** The mRNA level of CCR9 had a positive correlation with the mRNA level of CCL25. Each data point represents one colonic biopsy specimen in each panel, and each panel includes data from all colonic biopsy specimens in our experiments. Different symbols are used for UC patients and healthy controls in each panel. Data were analyzed by Pearson’s correlation coefficient.

A recent study demonstrated that NKT cells were originally determined based on their expression of NK-like markers such as CD161 (NK1.1) marker in UC [28]. Compared to the healthy controls, UC patients had mRNA levels of CD161, CCL25 and CCR9 that were significantly increased by 3-fold (0.11±0.04 vs. 0.04±0.02, *p* = 0.037), 31-fold (4.05±3.57 vs. 0.13±0.21, *p* = 0.001) and 46-fold (10.56±2.87 vs. 0.23±0.32, *p*<0.001), respectively (Figure 1A, B, C). The level of CD161 mRNA expression was significantly positively correlated with the levels of the CCL25 (r = 0.529, *p* = 0.008) and CCR9 (r = 0.900, *p*<0.001) mRNA expression (Figures 1D and E). As shown in Figure 1F, the level of CCR9 mRNA expression was significantly positively correlated with the levels of CCL25 mRNA expression (r = 0.528, *p* = 0.008) in all of the colonic biopsy specimens tested. These results showed that CD161, CCL25 and CCR9 expression levels were remarkably increased in UC patients and suggest that CCL25/CCR9 interactions have a positive correlation with NKT cells in the pathogenesis of UC.

### Oxazolone Induce Acute Forms of UC-like Colitis in BALB/c Mice

To examine the correlation between CCL25/CCR9 interactions and NKT cells, we used oxazolone-induced colitis in mice. Mice treated with oxazolone developed a rapid-onset colitis marked by weight loss and reduced survival rate. Mice were evaluated daily to assess the DAI (Table 1).

**Figure 2:**
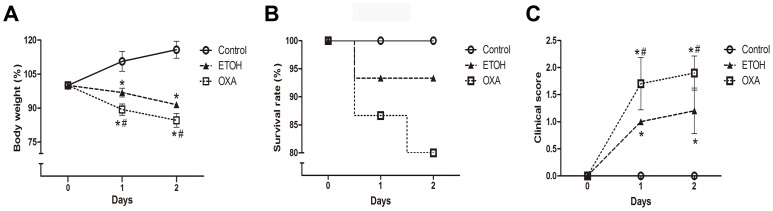
Changes in body weight, survival rate and clinical scores during oxazolone-induced colitis. **A.** The OXA mice lost the more body weight on day 2 compared to the ETOH mice. **B.** An 80% survival rate was observed in the OXA mice. **C.** The clinical scores of the OXA mice were significantly increased compared to the ETOH mice. n = 15 per group, *p<0.05 versus the control mice, and ^#^ *p*<0.05 versus the ETOH mice.

The difference in body weight for each mouse was determined daily by comparing the current weight with the weight on day 0. On day 1 and day 2, the body weights of the ETOH mice were significantly decreased by 12% (96.87±1.96 vs. 110.61±4.41%, *p*<0.001) and 21% (91.53±0.90 vs. 115.76±3.77%, *p*<0.001), respectively, compared to the control mice. The OXA mice had lost approximately 8% (89.37±2.46 vs. 96.87±1.96%, *p*<0.001) of their weight by day 1 and 9% (84.64±3.02 vs. 91.53±0.90%, *p*<0.001) on day 2 compared to the ETOH mice (Figure 2A). As shown in Figure 2B, the numbers of dead mice were 0 (0/15), 1 (14/15) and 3 (12/15) for the control mice, ETOH mice and OXA mice, respectively. The stool consistency, hematochezia and rectal bleeding of the mice were monitored beginning on day 0. As shown in Figure 2C, the ETOH mice and OXA mice had significantly elevated clinical scores compared to the control mice. Compared to the ETOH mice, the clinical scores of the OXA mice increased significantly by 1.7-fold (1.70±0.48 vs. 1.00±0.00, *p*<0.001) and 1.6-fold (1.90±0.32 vs. 1.20±0.42, *p*<0.001) on day 1 and day 2, respectively. It is clear that the body weight loss, mortality rates and clinical scores for the OXA mice were more than for the ETOH mice.

### The Macroscopic Clinical Signs and MPO Activity during Oxazolone-induced Colitis

**Figure 3:**
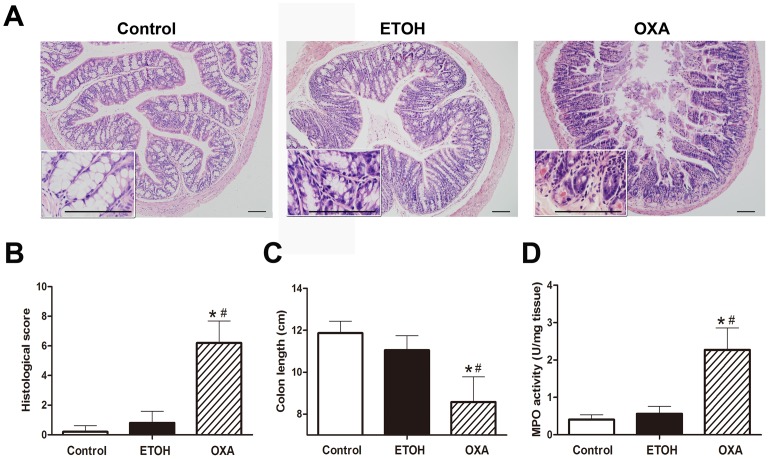
Changes in histological scores, colon length and MPO activity during oxazolone-induced colitis. **A.** Tissue slices were inspected by a certified pathologist, and the inflammation severity of colonic tissues from surviving mice in each group was scored and documented. Cross-sections represent the transverse colon. Image magnifications are 100-fold and 400-fold. Scale bar, 100 µm. **B.** The histological scores were higher in the OXA mice during colitis. **C.** The colon length of the OXA mice was significantly shorter than the ETOH mice. **D.** The MPO activity was significantly increased in the OXA mice compared to the ETOH mice. n = 10 per group, *p<0.05 versus the control mice, and ^#^ *p*<0.05 versus the ETOH mice.

The colons of the OXA mice showed histologic features similar to those of human UC, including crypt hyperplasia, crypt loss, cryptitis and neutrophil infiltration and mononuclear cells into the lamina propria. These histologic features were less evident in the ETOH mice (Figure 3A). Consistent with these findings, the histological scores were elevated in the OXA mice compared with the ETOH mice. The data are presented in Figure 3B (OXA, 6.20±1.48 vs. Control, 0.20±0.42, *p*<0.001; OXA, 6.20±1.48 vs. ETOH, 0.80±0.79, *p*<0.001).

The shortening of the colon that occurs due to ulceration and inflammation is an indicator of colitis severity in colitis mice [29]. As shown in Figure 3C, we observed shorter colons in the OXA mice than in the ETOH mice (8.58±1.20 vs. 11.05±0.70 cm, *p*<0.001). However, there was no significant reduction in colon length in the ETOH mice (11.05±0.70 vs. 11.87±0.57 cm, *p* = 0.067) compared to the control mice. In the OXA mice, MPO activity was significantly increased by 6-fold (2.27±0.59 vs. 0.40±0.13 U/mg tissue, *p*<0.001) compared with the control mice. Additionally, MPO was significantly increased by 4-fold (2.27±0.59 vs. 0.56±0.20 U/mg tissue, *p*<0.001) in OXA mice compared with the ETOH mice (Figure 3D). These results suggested that the OXA mice had acute UC-like colitis, which was determined based on histological appearance, histological scores, colon length and MPO activity.

### Increased Expression Levels of NK1.1, CCL25 and CCR9 are Found in Oxazolone-induced Colitis

**Figure 4:**
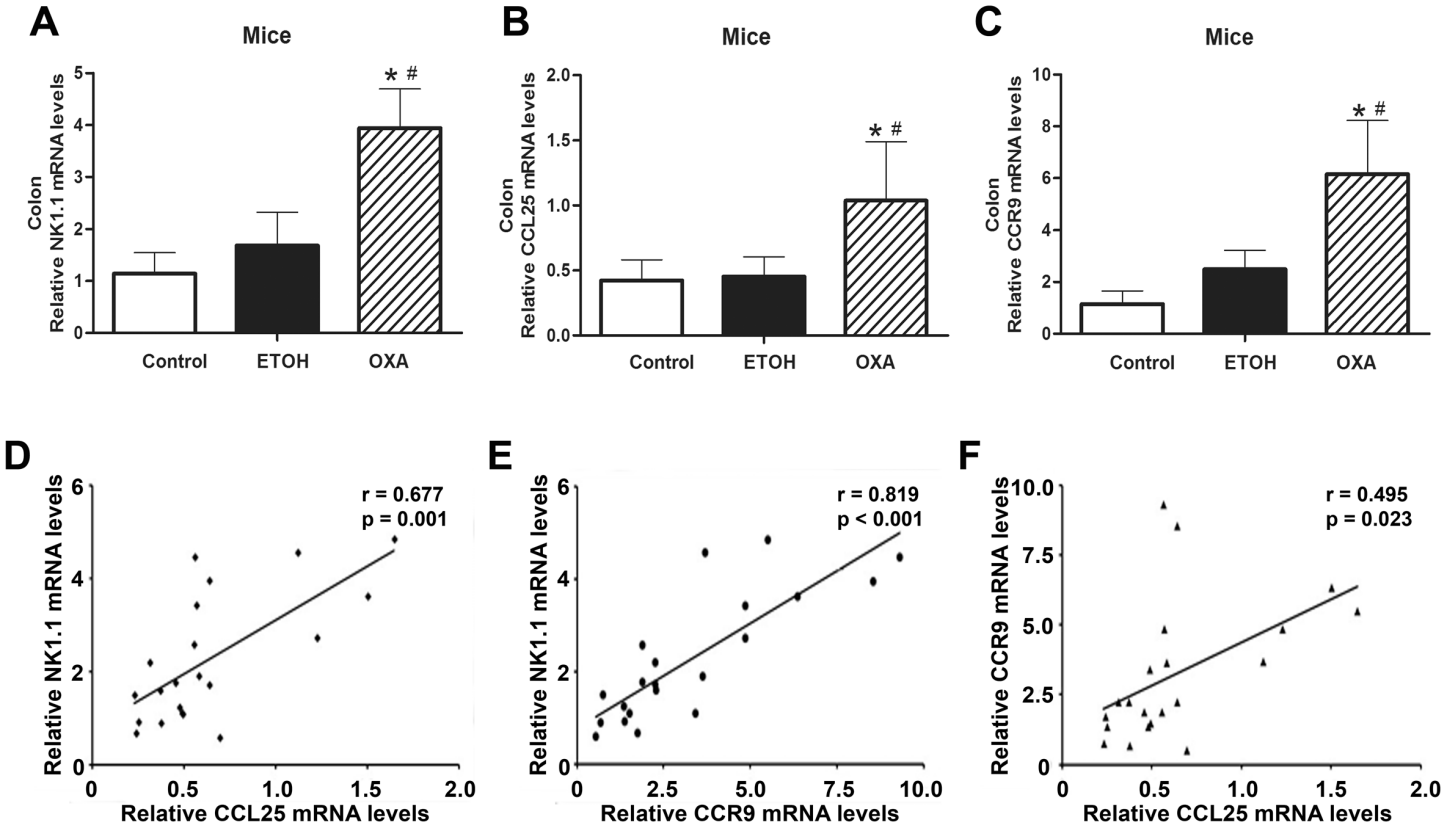
Expression levels of NK1.1, CCL25 and CCR9 during oxazolone-induced colitis. The mRNA levels of NK1.1 (**A**), CCL25 (**B**) and CCR9 (**C**) were significantly increased in the OXA mice compared to the ETOH mice. n = 10 per group, *p<0.05 versus the control mice, and ^#^ *p*<0.05 versus the ETOH mice. **D and E.** NK1.1 mRNA expression had positive correlations with CCL25 mRNA and CCR9 mRNA expression. **F.** The mRNA expression of CCR9 was significantly positively correlated with CCL25 mRNA expression. Each data point represents one colonic biopsy specimen in each panel, and each panel includes data from all colonic biopsy specimens in our experiments. Data were analyzed by Pearson’s correlation coefficient.

A low-level of expression for CCL25 and CCR9 has been detected in the large intestine [30]. To determine whether colitis affects the expression levels of NK1.1, CCL25 and CCR9, we analyzed the mRNA expression levels using qPCR. Compared to the control mice, the expression levels of NK1.1, CCL25 and CCR9 in OXA mice were significantly increased by 3-fold (3.94±0.75 vs. 1.14±0.40, *p*<0.001), 2-fold (1.04±0.45 vs. 0.42±0.16, *p* = 0.001) and 5-fold (6.15±2.07 vs. 1.15±0.50, *p*<0.001), respectively. As shown in Figure 4A, B, C, the expression levels of NK1.1, CCL25 and CCR9 were all increased by 2-fold (3.94±0.75 vs. 1.68±0.64, *p*<0.001; 1.04±0.45 vs. 0.45±0.15, *p*<0.001; 6.15±2.07 vs. 2.49±0.73, *p*<0.001) compared to the ETOH mice. However, the expression levels of NK1.1, CCL25 and CCR9 were not increased in the ETOH mice (1.68±0.64 vs. 1.14±0.40, *p*>0.05; 0.45±0.15 vs. 0.42±0.16, *p*>0.05; 2.49±0.73 vs. 1.15±0.50, *p*>0.05) compared to the control mice. As shown in Figures 4D and E, the level of NK1.1 mRNA expression was significantly positively correlated with the levels of the CCL25 (r = 0.677, *p* = 0.001) and CCR9 (r = 0.819, *p*<0.001) mRNA expression in all of the colonic biopsy specimens tested. The level of CCR9 mRNA expression was significantly positively correlated with the level of CCL25 mRNA expression (r = 0.495, *p* = 0.023) (Figures 4F). These results show that NK1.1, CCL25 and CCR9 expression levels increased in the OXA mice, suggesting that CCL25/CCR9 interactions are correlated with NKT cells during colitis.

### Production of Pro-inflammatory Cytokines during Oxazolone-induced Colitis

**Figure 5:**
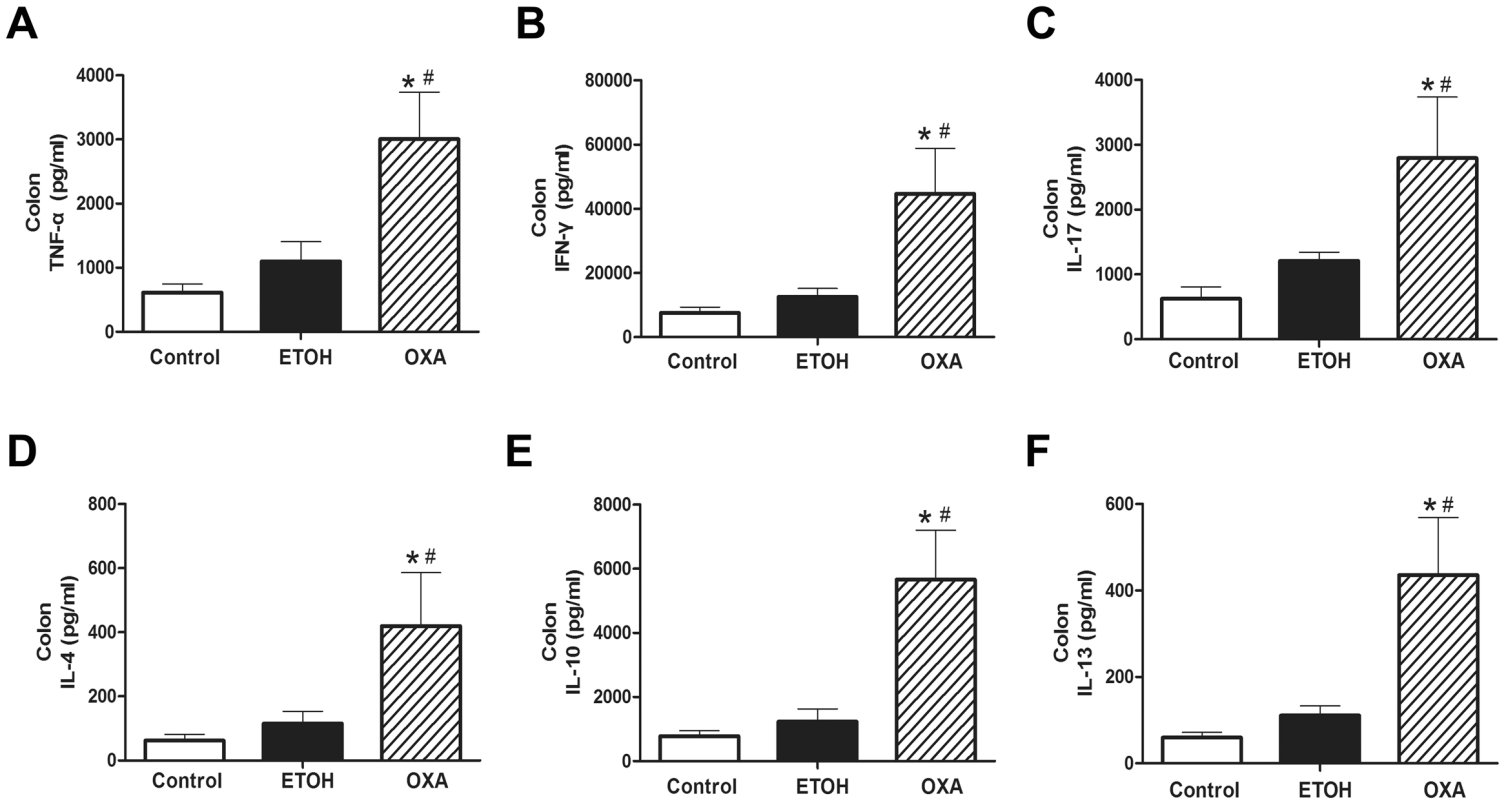
Production of TNF-α, IFN-γ, IL-17, IL-4, IL-10 and IL-13 during oxazolone-induced colitis. The levels of TNF-α (**A**), IFN-γ (**B**), IL-17 (**C**), IL-4 (**D**), IL-10 (**E**) and IL-13 (**F**) were significantly increased in the OXA mice compared to the ETOH mice. n = 10 per group, *p<0.05 versus the control mice, and ^#^ *p*<0.05 versus the ETOH mice.

To assess tissue inflammation, we analyzed the Th1/Th2/Th17 cytokines in intestinal tissue homogenates. As shown in Figure 5, the production of TNF-α, IFN-γ, IL-17, IL-4, IL-10 and IL-13 in OXA mice was significantly increased compared to the control and ETOH mice.

The production of TNF-α in OXA mice was increased by 5-fold (3003.79±732.95 vs. 613.23±133.16 pg/ml, *p*<0.001) and 3-fold (3003.79±732.95 vs. 1099.23±306.68 pg/ml, *p*<0.001), compared to the control and ETOH mice, respectively (Figure 5A). In the OXA mice, IFN-γ was increased by 6-fold (44637.53±14115.58 vs. 7543.80±1819.68 pg/ml, *p*<0.001) and 4-fold (44637.53±14115.58 vs. 12593.29±2589.37 pg/ml, *p*<0.001), compared to the control and ETOH mice, respectively, (Figure 5B). However, IL-17 was increased by 4-fold (2794.40±945.04 vs. 623.94±185.79 pg/ml, *p*<0.001) and 2-fold (2794.40±945.04 vs. 1209.65±134.73 pg/ml, *p*<0.001), respectively, compared to the control and ETOH mice (Figure 5C). Oxazolone strongly enhanced the production of IL-4, IL-10 and IL-13. The levels of IL-4, IL-10 and IL-13 were all increased by 7-fold (419.78±166.71 vs. 62.12±19.35 pg/ml, *p*<0.001; 5662.00±1544.00 vs. 773.65±179.00 pg/ml, *p*<0.001; 435.97±133.24 vs. 59.76±12.18 pg/ml, *p*<0.001) compared to the control mice, and by 4-fold (419.78±166.71 vs. 114.72±39.20 pg/ml, *p*<0.001; 5662.00±1544.00 vs. 1259.74±400.02 pg/ml, *p*<0.001; 435.97±133.24 vs. 110.72±22.25 pg/ml, *p*<0.001) compared to the ETOH mice (Figure 5D, E, F). These results indicated the levels of IL-4, IL-10 and IL-13 in the OXA mice were remarkably high compared to the ETOH mice, while the levels of TNF-α, IFN-γ and IL-17 changing little.

### Highly Increased Frequency of Colonic iNKT Cells and CCR9 Expression during Oxazolone-induced Colitis

**Figure 6:**
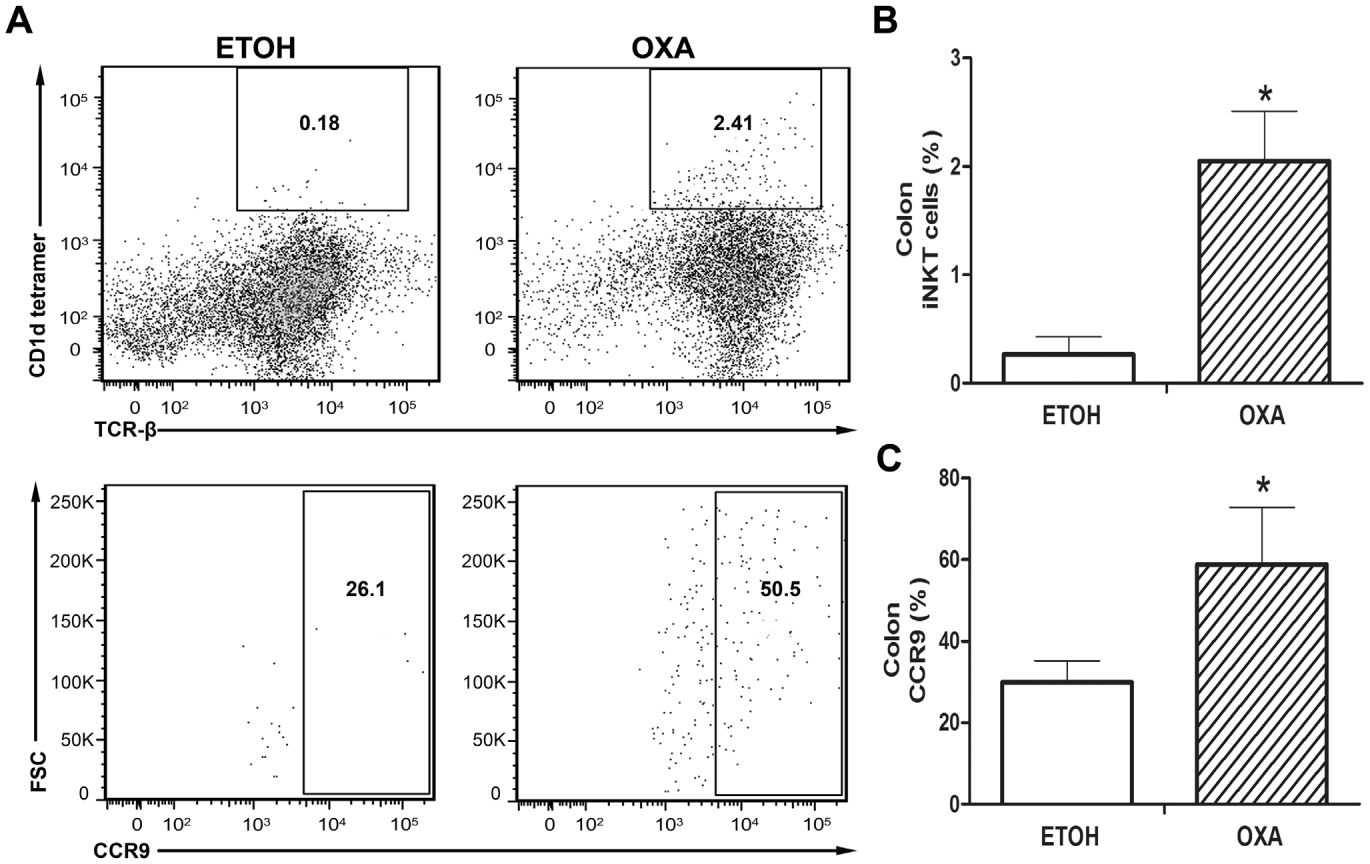
Frequency of colonic iNKT cells and CCR9 expression during oxazolone-induced colitis. The recruitment of iNKT cells and CCR9 expression on iNKT cells in LPL populations extracted from the colon of mice in three independent experiments by flow cytometry. **A.** The frequency of iNKT cells and CCR9 expression on iNKT cells in the OXA mice and ETOH mice. **B.** The percentage of iNKT cells was significantly increased in the OXA mice. **C.** Increased expression of CCR9 on iNKT cells was observed in the OXA mice. n = 3 per group, *p<0.05 versus the ETOH mice.

We investigated the recruitment of iNKT cells and expression of CCR9 by flow cytometry in LPL populations extracted from the colon of mice in three independent experiments. We observed that iNKT cells (TCRβ^+^ α-GalCer-loaded CD1d tetramer^+^ cells) were recruited in the colons of OXA mice (2.41%) and ETOH mice (0.18%) (Figure 6A). As shown in Figure 6A, CCR9 expression by the colon iNKT cells of OXA mice (50.5%) was increased compared to the colon iNKT cells of ETOH mice (26.1%). The percentage of iNKT cells was significantly increased by 8-fold (2.05±0.46 vs. 0.26±0.16%, *p*<0.001) in the OXA mice compared to the ETOH mice (Figure 6B). As shown in Figure 6C, the CCR9 expression levels on iNKT cells were higher in the OXA mice (2-fold, 58.87±14.01 vs. 29.90±5.32%, *p* = 0.001) compared to the ETOH mice. Interestingly, CCR9 expression on iNKT cells and the levels of iNKT cells were significantly increased during colitis. These results suggested CCL25/CCR9 interactions might promote the induction of iNKT cells during colitis.

### Highly Increased Chemotaxis of Colonic iNKT Cells Towards CCL25 during Oxazolone-induced Colitis

**Figure 7:**
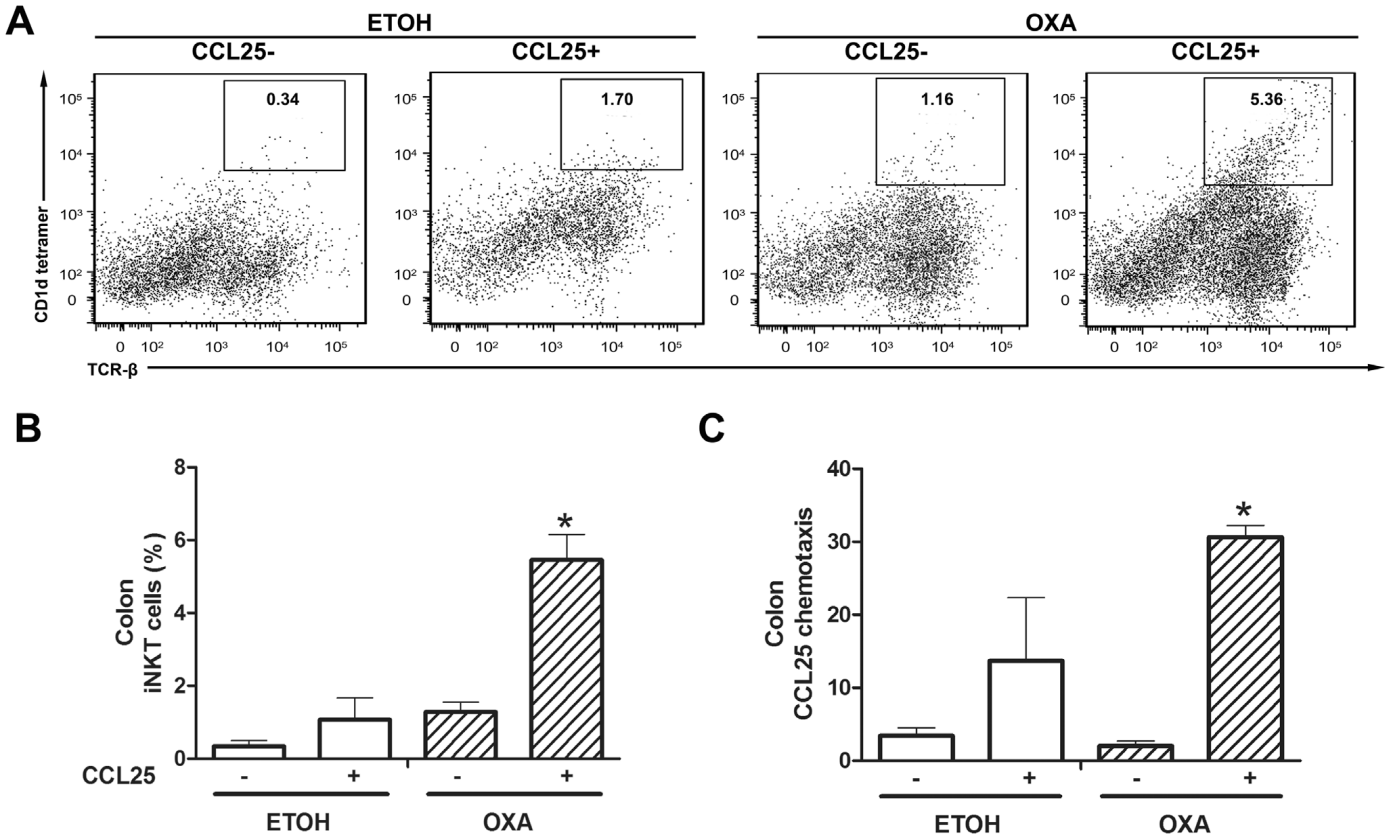
Chemotaxis of colonic iNKT cells towards CCL25 during oxazolone-induced colitis. The chemotaxis of colonic iNKT cells toward CCL25 was evaluated by a chemotaxis assay. **A.** After CCL25 treatment, the percentage of iNKT cells was determined in the LPLs from the OXA and ETOH mice. **B.** After chemotaxis to CCL25, the percentage of iNKT cells was significantly increased in the LPLs from the OXA mice. **C.** Colonic CCL25 chemotaxis was significantly increased in the LPLs from the OXA mice. n = 3 per group, *p<0.05 versus the ETOH mice.

To analyze the functionality of the chemokine receptors expressed by iNKT cells, we performed a chemotaxis assay to determine the ability of iNKT cells to migrate toward CCL25 (500 ng/ml). As shown in Figure 7A, the percentages of iNKT cells specifically migrating to CCL25 were increased in the LPLs from both ETOH and OXA mice. After chemotaxis to CCL25, the percentage of iNKT cells was increased by 4-fold (5.45±0.70 vs. 1.29±0.27%, *p* = 0.029) in the LPLs from OXA mice (Figure 7B). More iNKT cells migrated toward the chamber containing recombinant CCL25 than to the compartment without CCL25 (30.64±1.61 vs. 2.02±0.65, *p*<0.001) in the OXA mice (Figure 7C). These results suggested CCL25/CCR9 interactions might promote iNKT cell migration to the colon during colitis.

## Discussion

Although the exact etiology of IBD is unclear, it is know that altered immunological functions can contribute to the mucosal inflammation of the intestinal tract [31]. iNKT cell development is thymus dependent, but iNKT cells are found in the liver, spleen, lymph nodes and intestines [12]. iNKT cells are an important immunoregulatory population of lymphocytes able to polarize the immune response [12]. A murine model of oxazolone-induced colitis is associated with CD1d-dependent production of Th2 cytokines involved in mucosal inflammation [4], [32].

In the present study, we observed that CD161, CCL25 and CCR9 expression levels were significantly increased in the UC patients. We used a murine model of oxazolone-induced colitis to investigate the potential regulatory roles of CCL25/CCR9 interactions in NKT cells and the underlying mechanisms involved. We found that the expressions of NK1.1, CCL25 and CCR9 were significantly increased during oxazolone-induced colitis. Importantly, we demonstrated that human UC and UC-like colitis upregulated the expression of NKT, CCL25 and CCR9 in both humans and mice.

Chemokines are small heparin-binding proteins that play roles in multiple biological processes, including leukocyte chemotaxis [33]. Most chemokines can recognize several receptors, and one receptor binds many chemokines. However, the CCR9 receptor only binds CCL25 [34]. The combination of chemokine receptors expressed on the cell surface provides an “address” for leukocyte migration to different sites [35]. CCL25/CCR9 interaction is involved in gut-specific migration of leukocytes, suggesting this chemokine/receptor pair is non-promiscuous [36]. Wermers et al. recently demonstrated that chemokine CCL25 could induce Tregs to migrate to the intestine in chronic ileitis through its receptor CCR9 [37]. Although the induction of regulatory molecules occurs via common pathways, little is known about the roles of CCL25/CCR9 interactions in iNKT trafficking and regulation of immune responses in the colon. We observed significantly increased CCR9 expression on iNKT cells, an increased iNKT population and increased iNKT chemotaxis. It is reasonable to speculate that CCL25 signaling through CCR9 might induce iNKT cells to migrate into the colon during human UC and UC-like colitis.

NKT cells have complicated biological functions and can rapidly produce large amounts of Th1, Th2 and regulatory cytokines [38]. It has been shown that iNKT cells play an important role in the pathogenesis of UC and secrete large amounts of pro-inflammatory cytokines such as IL-4 and IL-13 upon activation [10], [11]. Mouse NKT cells have been recognized as immunosuppressive cells that produce Th2 cytokines or IL-10 [39]. Our interpretation that the results are a consequence of a highly inflammatory response in OXA mice is supported by our data in two ways. First, there was a pronounced accumulation of iNKT cells. Second, there was increased expression of the pro-inflammatory cytokines, especially IL-4, IL-10 and IL-13. The increased expression of these pro-inflammatory cytokines has also been described for human UC [40].

We demonstrated significantly increased CCR9 expression on iNKT cells, an expanded iNKT population and increased chemotaxis of iNKT cells during oxazolone-induced colitis. It is our hypothesis that CCL25/CCR9 interactions promote the induction and function of iNKT cells during oxazolone-induced colitis. However, it is yet to be tested the role of CCL25/CCR9 interactions in iNKT cells migrating into the colon. CCR9- or CCL25-deficient animals or antibody-mediated neutralization of these factors are needed in future studies to definitively define the function and mechanism of CCL25/CCR9 interactions in the regulation of iNKT cells during oxazolone-induced colitis.

In conclusion, this study has shown that CCL25/CCR9 interactions may promote the induction and function of iNKT cells during oxazolone-induced colitis. These findings expand our understanding of the underlying mechanism of CCL25/CCR9 interactions in the regulation of iNKT cells during human UC and UC-like colitis. This study has provided further evidence that CCR9-inhibitors could be a potential therapeutic strategy for patients with UC.
